# Phycoremediation Potential of Salt-Tolerant Microalgal Species: Motion, Metabolic Characteristics, and Their Application for Saline–Alkali Soil Improvement in Eco-Farms

**DOI:** 10.3390/microorganisms12040676

**Published:** 2024-03-28

**Authors:** Huiying Chen, Siteng Yu, Ze Yu, Meng Ma, Mingyan Liu, Haiyan Pei

**Affiliations:** 1 School of Environmental Science and Engineering, Shandong University, Qingdao 266237, China; chenhuiying07@163.com (H.C.); y820099450@163.com (S.Y.); 15001956061@163.com (M.M.); 17853728873@163.com (M.L.); 2 Department of Environmental Science and Engineering, Fudan University, Shanghai 200433, China; yz201512044@126.com; 3 Shandong Provincial Engineering Center on Environmental Science and Technology, Jinan 250061, China; 4 Institute of Eco-Chongming (IEC), Shanghai 202162, China

**Keywords:** microalgae, salt-affected soil, nylon screen, biomass production, monosodium glutamate residue, soil improvement

## Abstract

Microalgae have great potential for remediating salt-affected soil. In this study, the microalgae species *Coelastrella* sp. SDEC-28, *Dunaliella salina* SDEC-36, and *Spirulina subsalsa* FACHB-351 were investigated for their potential to rehabilitate salt-affected soils. Nylon screens with optimal aperture sizes and layer numbers were identified to efficiently intercept and harvest biomass, suggesting a correlation between underflow capability and the tough cell walls, strong motility, and intertwining characteristics of the algae. Our investigations proved the feasibility of incorporating monosodium glutamate residue (MSGR) into soil extracts at dilution ratios of 1/200, 1/2000, and 1/500 to serve as the optimal medium for the three microalgae species, respectively. After one growth period of these three species, the electrical conductivities of the media decreased by 0.21, 1.18, and 1.78 mS/cm, respectively, and the pH remained stable at 7.7, 8.6, and 8.4. The hypotheses that microalgae can remediate soil and return profits have been verified through theoretical calculations, demonstrating the potential of employing specific microalgal strains to enhance soil conditions in eco-farms, thereby broadening the range of crops that can be cultivated, including those that are intolerant to saline–alkali environments.

## 1. Introduction

Agricultural sustainability is crucial for ensuring global food security in the coming decades [1,2], and soil fertility is a key determinant of agricultural productivity [3]. However, the intensive use of chemical fertilizers and pesticides in recent decades has degraded soil fertility and has also caused the widespread formation of saline soils [4], posing significant constraints for the sustainable development of global agriculture. Enhancing the utilization of saline soils could help alleviate food insecurity [5]. Moreover, carbon neutrality has emerged as a critical long-term strategy of both the UN Climate Change Conference (COP26) and China’s Peak CO_2_ Action Plan [6].

Conventional physical methods to leach soluble salts from the soil with fresh water are water-intensive and nutrient-depleting. Chemical methods that use amendments such as flue gas desulfurization gypsum can reduce soil salinity by adsorbing ions, but they may also introduce new ions and cause secondary pollution [7]. Biological methods that exploit phytoremediation and microbial remediation are ecological, but they often need to be combined with other methods to improve effectiveness. Microalgae are photosynthetic microorganisms that convert carbon, water, light, and salts from the inorganic environment into biomass and nutrients. Soil is an important habitat for algal evolution [2,8]. Microalgae have developed a series of unique and valuable characteristics that hold great promise for their use in saline soil remediation and nitrogen and carbon fixation [9]. Researchers in India first reported in 1939 that nitrogen-fixing cyanobacteria were used for fertilizing fields [10]. Since then, numerous studies have shown that soil inoculation with nitrogen-fixing cyanobacteria resulted in significant increases in soil nitrogen content and crop yields. Microalgae can also enhance soil organic carbon by photosynthesizing atmospheric CO_2_ and producing nutrient-rich biomass [11]. Overall, algae fix about 9.5 × 10^10^ tons of CO_2_ per year, accounting for 47.5% of the net photosynthetic output of the world, and play an important role in energy conversion and cycling processes [12,13].

Significant benefits of microalgae on soils are effected through the secretion of a diverse range of bioactive substances, which progressively enhance the structure and composition of saline soils. The primary function of microalgae as soil conditioners appears to focus on improving nutrient use efficiency rather than simply replacing the nutrients [14]. Rao and Burns [15] have studied the effects of cyanobacterium inoculation on the surface properties of brown soils and found significant increases in soil polysaccharides, dehydrogenase, urease and phosphatase activities, and soil water content. Wang et al. [16] have reported that algal cells decompose to organic fertilizer when they die, thereby increasing crop yield and quality.

There are currently two main ways in which microalgae are used to improve saline soils. One is to apply microalgae as algal fertilizers in combination with phytoremediation, which involves a complex selection of microalgae and plants and requires large amounts of microalgal biomass and freshwater [17]. The other is to stimulate the growth of native soil microalgae by irrigation or rainfall, which produces extracellular polymers that adsorb salts and promote soil aggregate stability; however, this method is unsuitable for non-irrigated areas and areas with short rainy seasons [18]. Although microalgae are important for saline–alkali land improvement, their effectiveness in reducing soil salinity is still debated. Some researchers have argued that the release of adsorbed and enriched salt ions after the death of microalgae leads to an unstable enhancement effect if microalgae are not harvested [6]. Despite these challenges and the ongoing debate surrounding the efficiency of microalgae in remediating saline–alkali soils, a significant hurdle to overcome lies in the dearth of suitable algal strains with high salt tolerance. Insufficient algal germplasm resources with adequate salt tolerance significantly impede the broad application and development of microalgal technology for saline–alkali soil remediation. Indeed, while acknowledging the potential roles that higher plants may play in soil remediation efforts [19], this study’s primary focus is on the unique advantages offered by microalgae for addressing salinity issues. Unlike some terrestrial plant species that might require more extended periods to establish and exhibit varying degrees of salt tolerance, microalgae demonstrate rapid growth rates [20], high tolerance to extreme conditions, and efficient nutrient cycling capabilities [21]. Their ability to sequester carbon dioxide and produce biomass even in saline environments makes them a promising candidate for innovative restoration strategies.

Among the commonly recognized salt-tolerant microalgae species are Chlorophyta (e.g., *Chlorella*, *Scenedesmus*, and *Dunaliella*) and *Cyanobacteria* (e.g., *Spirulina*, *Nostoc*, *Oscillatoria*, and *Anabaena*), yet they exhibit substantial interspecific differences in desalination capacity [22]. The current strain diversity falls short of meeting practical demands, and reports of specialized strains tailored for saline–alkali soil restoration are infrequent. This scarcity is largely due to the prevalent reliance on blind screening methods using pot experiments to assess their restorative effects, which can be time-consuming, yield low success rates, and lack reproducibility as well as a reliable standard for assessing high-quality strains. In light of these limitations and the pressing need for effective and practical solutions, our research focuses on specific indigenous and commonly known salt-tolerant strains. Therefore, considering these issues, we specifically chose two indigenous green algae species—*Coelestrella* sp. SDEC-28 and *Dunaliella salina* SDEC-36, isolated from saline–alkali soil, along with the typically salt-tolerant blue-green alga *Spirulina subsalsa* FACHB-351 for our study. These selected strains all demonstrate robust growth and reproduction capabilities under high-salinity conditions and possess strong adaptability and potential for remediating saline–alkali soils. Moreover, this investigation also delves into comparing the activity and adaptability of native versus non-native microalgae when cultivated in saline–alkali environments.

A microalgal eco-farm on salt-affected soil was proposed in our previous study [6], wherein a nylon screen was used to prevent, as much as possible, microalgae from entering the soil. The screens would be suspended to separate the microalgal biomass from the liquid, and the screens can be conveniently harvested by scraping, avoiding the energy-intensive processes of centrifugation or filtration. This means that a portion of the salt in the soil is carried out with the harvest of microalgae, thereby achieving desalination and alkalinity reduction. The small portion of microalgae that underflowed the boundaries of the screen and entered the soil elevated the organic carbon content of the soil. This microalgal eco-farm has achieved both in situ soil improvement and microalgal cultivation. However, the majority of salt-affected soils are too barren and need extra nutrients, so supplements must include high levels of nutrients while being free of heavy metals and toxicity. Our studies have demonstrated that monosodium glutamate residue (MSGR) is a satisfactory nutrient for many microalgal species, as microalgae can assimilate nutrients such as nitrogen and phosphorus [23,24]. However, comprehensive research is required on the selection of screens and supplementary nutrients.

To address the current problems, the main goals comprised: (1) investigating the effects of aperture sizes and the number of layers of screens on microalgal biomass production; (2) combining MSGR with soil extracts as media at different addition ratios to explore the feasibility of quickly gaining higher biomass concentrations; (3) analyzing the changes in the physical and chemical properties of the culture medium before and after cultivation; and (4) obtaining predicted values of the microalgae’s assimilation of nutrients in the soil and verifying the feasibility of constructing microalgal eco-farms.

## 2. Materials and Methods

### 2.1. Preparation of soil and Soil Extract

Soil samples were taken from Dongying (Shandong province, China). Then, the soil samples were pre-treated. The soil samples were air-dried naturally, and the gravel and other debris were removed. After the dried soil samples were manually ground using a wooden hammer, they were filtered with a 20-mesh nylon screen (0.9 mm aperture size) and mixed evenly. To determine the initial physical properties of the soil, these soil samples were mixed in a soil–water ratio of 1:2.5 (*m*_soil_/*m*_water_), and the pH was determined to be 8.1. The soil was additionally mixed in a soil–water ratio of 1:5 (*m*_soil_/*m*_water_), yielding an electrical conductivity (EC) of 6.9 mS/cm and a salinity of 0.38%.

For the preparation of the soil extract (SE), 100 g of pre-treated soil was measured and poured into a 1 L conical flask; 500 mL of deionized water was added into the conical flask and *Parafilm* was used to seal it. The conical flask was then placed on a fully thermostatic oscillator, shaken at 180 to 200 rpm for 3 h at 25 ± 1 °C, and then left to stand. The supernatant was passed through a 0.45 μm filter membrane to obtain saline–alkali soil extract. The values of these three physical indexes (pH, conductivity, and salinity) in the soil extract were not significantly different from those of the original soil.

### 2.2. Microalgal Species and Monosodium Glutamate Residue

Three microalgal species were used in the experiments. The microalgal species *Coelastrella* sp. SDEC-28 and *Dunaliella salina* SDEC-36 were previously isolated from 0–20 cm topsoil in saline–alkali soil in Dongying. *Spirulina subsalsa* FACHB-351 was purchased from the Freshwater Algae Culture Collection of the Institute of Hydrobiology (FACHB), which is China’s specialized institution dedicated to the preservation, utilization, and management of freshwater algal strains resources (Swing by their website http://algae.ihb.ac.cn/ to learn more, accessed on 1 January 2024). Among them, *Coelastrella* sp. and *Dunaliella salina* belong to the phylum *Chlorophyta*, and *Spirulina subsalsa* belongs to the phylum *Cyanobacteria*. Cultivations of *Coelastrella* sp. were carried out in photobioreactors in BG11 medium at 25 ± 1 °C under 45 µmol/m^2^/s of 24 h light to obtain healthy seed cells for further studies. *D. salina* and *S. subsalsa* were cultivated under the same conditions in the SP medium (see Supplementary Materials).

MSGR was collected from Shandong Linghua Monosodium Glutamate Co. (Jining, Shandong Province, China) and was characterized by its dark brown color, which stems from a high content of melanoidin-like substances. It contains abundant residual sugars, amino acids, (NH_4_)_2_SO_4_, various organic acids, and defoamers, among other compounds. MSGR is typically characterized by its high levels of Chemical Oxygen Demand, high Biological Oxygen Demand, elevated bacterial cell content, high concentrations of ammonia nitrogen, high concentrations of sulfate, and low pH [24], and presented the following nutrient characteristics: 55.66 ± 6.80 g/L total nitrogen (TN), 3.94 ± 0.36 g/L total phosphorus (TP), 51.01 ± 0.16 g/L ammonia nitrogen (NH_3_-N), and 190.4 ± 5.54 g/L chemical oxygen demand (COD). The collected MSGR was first filtered with eight layers of gauze to remove insoluble solids. The quality of samples from different batches did not vary by more than 10%.

### 2.3. Experimental Design

**Figure 1:**
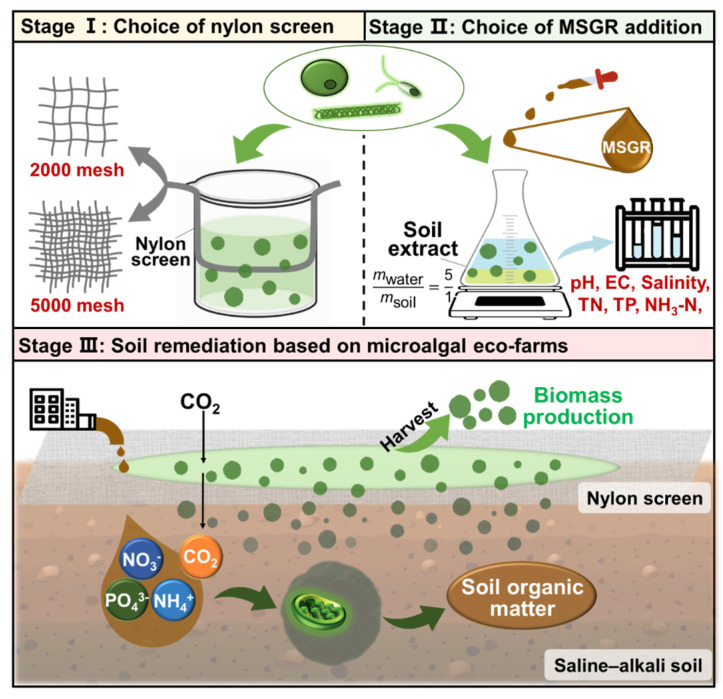
An innovative approach to soil remediation: A comprehensive strategy utilizing microalgal eco-farms. Stage Ⅰ–ⅠⅠⅠ: From selecting nylon screens and adding MSGR to implementing soil remediation through microalgal eco-farms.

The whole experimental process was divided into three stages, with the three types of microalgae mentioned in Section 2.2 as the research subjects (Figure 1). The first stage is to select screens with different aperture sizes and layers to cultivate microalgae. The purpose is to obtain the most suitable screen aperture and number of layers for each of the three types of microalgae. The second stage is to cultivate microalgae using MSGR in combination with prepared SE at different addition ratios. We measured the changes in physical and chemical properties of the media to simulate the impact of characterization on soil properties. We determined the optimal MSGR addition ratio for the three types of microalgae based on comprehensive biomass concentration, nitrogen, phosphorus utilization, and changes in physical indicators. In this experiment, our primary focus was on the operations of the two stages, and finally, according to the conditions selected in the previous two stages, we proceeded to simulate the positive impacts manifested as increases in soil organic matter content from carbon fixation and from the assimilation of nitrogen and phosphorus by microalgae underflowing the screen into the soil. To conclude, we verified the rationality of the construction of microalga-based ecological farms, as mentioned in the introduction, according to the above three experimental stages.

#### 2.3.1. Experiment 1—Choice of Screen Aperture and Number of Layers

The screen used to trap microalgae is made of nylon and polyester by mechanical blending, which has aperture sizes of 6.5 μm (2000 mesh) or 2.6 μm (5000 mesh). The respective prices of 2000 and 5000 mesh screens were USD 7.73 and USD 16.16 per square meter.

A total of 200 mL of standard culture medium was added to a beaker. The two types of screens were cut into appropriate sizes and fixed in beakers using one, two, or three layers. The culture medium was divided into 100 mL upper and 100 mL lower. Microalgae at the end of the logarithmic growth period were recovered by centrifugation and washed with distilled water three times before being inoculated into the upper culture medium. The initial inoculation amount was about 0.2 g/L. The outside of the beaker was wrapped with aluminum foil to ensure a dark environment, and the top was sealed with a preservative film to reduce evaporation. The beaker without a screen was used as a control. The culture top illumination was maintained at 45 µmol/m^2^/s and the temperature was maintained at 25 ± 1 °C. The experiments were carried out in triplicate.

#### 2.3.2. Experiment 2—Choice of Wastewater Addition Ratio

Different ratios of MSGR to SE were made up as nutrient sources. Dilution ratios of MSGR to SE (*V*_MSGR_/*V*_SE_) were set based on the contents of nitrogen and phosphorus in the BG11 medium of *Coelastrella* sp. and the SP medium of *D. salina* and *S. subsalsa*. BG11 medium and SP medium were used as controls, respectively.

The 250 mL conical flasks sealed with *Parafilm* were used as the experimental containers. The microalgae at the end of the logarithmic growth period were centrifugally cleaned and inoculated into the above media. The culture system volume was 150 mL, and the initial biomass concentration was about 0.1 g/L. All conical flasks were held at 25 ± 1 °C under 45 µmol/m^2^/s of 24 h light. During cultivation, the flasks were shaken twice each day. The experiments were carried out in triplicate.

### 2.4. Analysis of Algal Growth

Chlorophyll *a* is a key pigment in algal cells, playing a significant role in cell growth and metabolism, with its peak absorption at around 680 nm. Consequently, the light absorbance measured at 680 nm is directly proportional to changes in cell count in most unicellular organisms, thereby characterizing algal cell growth [25,26]. Hence, the daily measurement of *OD*_680_ allows for monitoring the growth of microalgae, and the daily biomass concentration (BC, g/L) was calculated through Equations (1) and (2):

(1) $${\mathrm{BC}}_{\mathrm{sdec}\text{-}28}\;(g/L)=0.3757\;{\mathrm{OD}}_{680}+0.0139,\;r^{2}=0.9989$$

(2) $${\mathrm{BC}}_{\mathrm{sdec}\text{-}36}\;(g/L)=0.4131\;{\mathrm{OD}}_{680}-0.0116,\;r^{2}=0.9953$$

where *BC*_sdec-28_ and *BC*_sdec-36_ are the respective biomass concentrations for *Coelastrella* sp. and *D. salina*.

The method to obtain the *S. subsalsa* cells was based on the work of Jiang et al. [15]. The biomass concentration can be calculated from Equation (3):

(3) $${\mathrm{BC}}_{\mathrm{FACHB}\text{-}351}\;(g/L)=(M_{1}-M_{0})/V$$

where *BC*_FACHB-351_ is the concentration of biomass for *S. subsalsa*. *M*_0_ (g) represents the weight of the self-sealing bag, *M*_1_ (g) represents the final mass of the bag and the *S. subsalsa* dry biomass, and *V* (L) represents the volume of all cultures.

The biomass productivity, *P*_b_ (mg/L/d) can be calculated based on Equation (4):

(4) $$P_{b}\;(\mathrm{mg}/L/d)=1000\times ({\mathrm{BC}}_{2}-{\mathrm{BC}}_{1})/(t_{2}-t_{1})$$

where *BC*_2_ and *BC*_1_ represent the biomass concentration on days *t*_2_ and *t*_1_, respectively.

The specific growth rate of microalgae in the logarithmic phase, *μ* (d^−1^) can be calculated according to Equation (5):

(5) $$\mu \;(d^{-1})=[\ln\;({\mathrm{BC}}_{2})-\ln\;({\mathrm{BC}}_{1})]/(t_{2}-t_{1})$$

where *BC*_2_ and *BC*_1_ represent the biomass concentration on days *t*_2_ and *t*_1_, respectively.

The concentration of photosynthetic pigments was measured by the method of dimethyl sulfoxide (DMSO) extraction [27]. The photosynthetic pigment concentrations were estimated using the following equations:

(6) $$\mathrm{Chlorophyll}\;a\;(\mathrm{mg}/L)=12.7A_{663}-2.69A_{645}$$

(7) $$\mathrm{Chlorophyll}\;b\;(\mathrm{mg}/L)=22.9A_{645}-4.68A_{663}$$

(8) $$\mathrm{Total}\;\mathrm{chlorophyll}\;(\mathrm{mg}/L)=20.2A_{645}+8.02A_{663}$$

(9) $$\mathrm{Carotenoids}\;(\mathrm{mg}/L)=A_{480}+0.114A_{663}-0.638A_{645}$$

where *A*_480_, *A*_645_, and *A*_663_ represent the absorbances of a sample at 480, 645, and 663 nm, respectively. Absorbances at 480, 645, and 663 nm were corrected for turbidity by subtracting absorbance at 750 nm if the supernatant was turbid after centrifugation.

### 2.5. Analysis of the Water Quality Index

The microalgal culture was centrifuged at 4000 rpm for 10 min, and the supernatant was filtered through a 0.45 µm membrane for the determination of pH, EC, salinity, TN, TP, and NH_3_-N. All the measurements were conducted based on Chinese state standard testing methods [28].

The average yield coefficient (AYC) (mg/g), and the assimilation rate (AR) (mg/L/d) were calculated from Equations (10) to (11):

(10) AYC (mg/g)=ΔC/ΔBC

(11) AR (mg/L/d)=ΔC/ΔT

where Δ*C* (mg/L) is the reduction in concentration due to the assimilation of nutrients (TN, TP, or NH_3_-N) during cultivation, and Δ*BC* (g/L) and Δ*T* (d) are the increases in the biomass concentration and culture time.

### 2.6. Statistical Analysis

The data were expressed as the mean ± standard deviation (SD) of the experiments in triplicate. SPSS software (version 26.0) was used to compare the parameters through Duncan’s test. A value of *p* < 0.05 was considered statistically significant.

## 3. Results and Discussion

### 3.1. Effects of Acreen Aperture Size and Number of Layers on Growth of Microalgae

**Figure 2:**
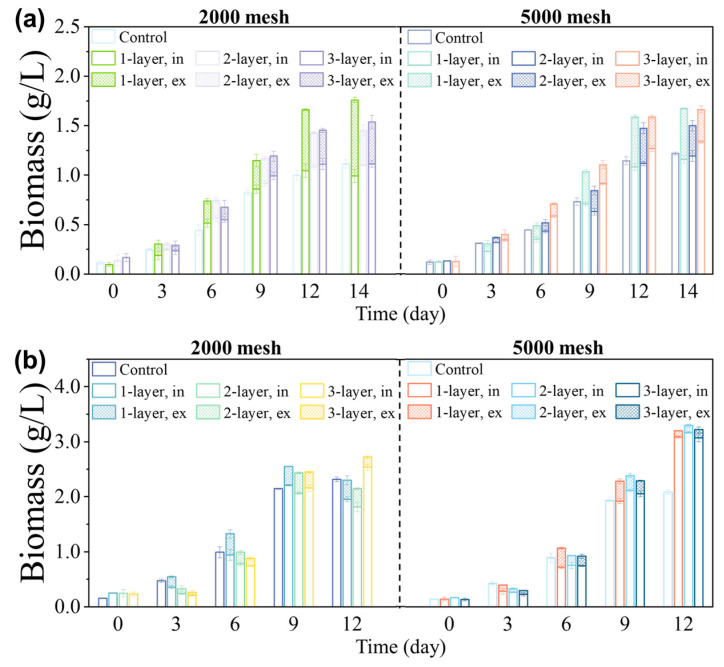
Choice of screen aperture and number of layers. The biomass concentrations of (**a**) *Coelastrella* sp. and (**b**) *D*. *salina* were cultured in different numbers of screen layers, with 2000 mesh or 5000 mesh screens. The microalgal strains were cultured using standard medium (*Coelastrella* sp. using BG11, and *D*. *salina* using SP). “Control” indicates the condition without any screen. The “1-layer”, “2-layer”, and “3-layer” labels indicate the use of 1, 2, and 3 layers of screens, respectively. The label “in” indicates the part inside the screens, and “ex” indicates the part that underflows the screens.

Figure 2a shows the growth of *Coelastrella* sp. inside nylon screens. As the culture time increased, the biomass concentrations both inside and outside the screen rose significantly, along with the proportion of biomass that had underflowed the screen. However, using a single layer of 2000 mesh screen resulted in a sharp increase in the underflow proportion on day 12. The reason may be the irregular movement and the action of gravity on *Coelastrella* sp. with thick and tough cell walls, resulting in the deformation and expansion of some holes in the screen, causing a large number of algal cells to escape into the external environment. The biomass concentration outside the screen grew rapidly, reducing harvest efficiency. When selecting 5000 mesh screens, the underflow proportion of each layer was essentially maintained below 30%. With a small mesh aperture, only a few microalgal cells escaped through the holes in the screens, while the rest formed arch-like shapes at holes, supporting the upper particles by the arch effect and maintaining stability. The analytic hierarchy process (AHP) was adopted to identify the best screen for each species. The criteria that can influence screen selection can be grouped into the biomass concentration within the screen, the underflow proportion, and the screen price. Each screen with a different aperture size and number of layers was considered as an alternative. The alternative with the highest score is the best. Three layers of 5000 mesh screen yielded a maximum score of 0.269 (Figure S2b), which was optimal for *Coelastrella* sp. cultivated on the soil (see Supplementary Materials).

Figure 2b shows the growth of *D. salina* using screens with different aperture sizes and numbers of layers. *D. salina* grew well, and its maximum specific growth rate during the exponential growth phase was not significantly different from that of the control group. The 5000 mesh screens performed significantly better than the 2000 mesh screens. For screens with larger aperture sizes (6.5 μm > 2.6 μm), increasing the number of layers was particularly beneficial. However, beyond a certain point, adding more layers did not noticeably reduce the loss rate. Multi-layer screens had a better ability to retain microalgal cells than single-layer screens. The microalgal cells that escaped from the first screen could still be captured by a subsequent screen, and those remaining on the filter layer could form an arch with the cells above them to prevent those cells from passing through. According to AHP analysis (see Supplementary Materials), the highest score of 0.241 was obtained when selecting a single layer of 5000 mesh screen (Figure S2b). Thus, to balance the biomass screened and the cost, this type of screen was optimal for *D. salina* cultivated on the soil. Moreover, the final biomass concentrations of the control groups without screens for *Coelastrella* sp. and *D. salina* were comparable to the experimental groups with screens, indicating that the screens used did not impair microalgal growth.

The filamentous shape and buoyancy of *S. subsalsa* cells caused uneven spatial distribution, which limited the use of spectral analysis tools for daily growth measurement. Thus, only the mass of dry microalgal cells at the final harvest could reflect the growth status. Using single-layer screens with a rating of 2000 mesh yielded the highest biomass concentration of 1.14 g/L above the screen (Figure S2a). Increasing the number of screen layers and decreasing the aperture size reduced the biomass concentration instead. This might be because *S. subsalsa* cells tangled and adhered to the screen, and more layers and smaller aperture sizes (6.5 μm > 2.6 μm) made them more tightly intertwined on the screen, affecting their ability to grow in clusters. Increasing the number of screen layers and decreasing the aperture size also led to a gradual decrease in the *S. subsalsa* underflow proportion. The filaments that wrapped around the screen wires blocked the holes, reducing underflow potential. Analysis for *S. subsalsa* (see Supplementary Materials)—similar to that for *Coelastrella* sp. and *D. salina*—yielded a score for the single-layer 2000 mesh screen (0.249) that was significantly higher than those of other alternatives (Figure S2b), so that configuration was chosen for cultivating *S. subsalsa* on the soil in subsequent experiments.

### 3.2. Relationship between the Microalgae’s Characteristics and Screen Properties

Microscope images of the three species before and after incubation with screens of varying aperture sizes are shown in Figure S3. The aperture size and shape of the screens remained largely unchanged after exposure to the three microalgal species. *Coelastrella* sp. and *D. salina* barely adhered to the screens, whereas *S. subsalsa* was evenly tangled on the surface of the screens, which significantly improved the retention capacity of screens.

Microalgae typically require filtration devices to separate them from media or other particles during harvesting [29]. The cell diameters of *Coelastrella* sp. ranged from 8 to 11 μm in BG11 culture and reached up to 15 μm during division. *D. salina* had algal cells that were typically 7 to 12 μm long, 3 to 8 μm wide, with two flagella that were 10 to 20 μm long. The algal cell bodies of *S. subsalsa* were 200 to 500 μm long and 5 to 10 μm wide. The pore sizes of the 2000 mesh and 5000 mesh screens used were 6.5 μm and 2.6 μm, respectively. However, microalgae could escape through nylon screen pores that were much smaller than their size during cultivation. There are some connections between microalgal characteristics and screen properties (Figure S4d).

A possible explanation for the passage of microalgae through tiny screen apertures is the deformability of the cell walls and membranes [30]. Most cyanobacteria and green algae have flexible cell walls and membranes. The cell wall and membrane can change shape in response to external stimuli. When microalgal cells try to pass through small pores, the cell wall stretches and the lipid bilayer of the cell membrane folds, allowing the cells to elongate and bend during movement. This allows them to move through screen pores with diameters smaller than their spherical shape. The ability of *Coelastrella* sp. to maintain cellular integrity even when stimulated by the addition of 4% salinity was due to its thick and tough cell wall (Figure S4a).

Microalgae with elongated shapes escape through screen pores more easily than spherical microalgae [30]. *S. subsalsa* is a microorganism with a natural microscopic helical structure that can pass through small pores easily due to its cylindrical shape and ability to twist and bend. Moreover, its tendency to grow in long filaments may help it to extend beyond the screen and break, allowing cells to escape (Figure S4c). However, it is also prone to intertwining, which greatly enhances the screen retention ability of *S. subsalsa*, making it feasible to minimize the underflow proportion while harvesting biomass using a single layer of 2000 mesh screen as described in Section 3.1.

*D. salina* lacks a rigid cell wall and is protected only by a thin plasma membrane with a mucous substance [31]. This allows the cell to squeeze through narrow openings without damaging the membrane and regain its original shape after passing through the pores due to its elasticity. *D. salina* has two long hairy appendages called flagella. Such flagella may also facilitate microalgae’s passage through micropores. They impart motility to *D. salina*, allowing the cells to move efficiently and directionally. Due to its powerful mobility, a ghosting effect caused by the rapid movement of *D. salina* was clearly visible under the microscope (Figure S4b). Having two flagella has several benefits for *D. salina*. These structures provide strong stability and control over movement direction and enables more complex movement patterns, which help the cells to navigate through intricate environments. Microalgae with flagella are more likely to cross small pores than those without flagella. As described in Section 3.1, *Coelastrella* sp. required 3-layer 5000 mesh screens, whereas *D. salina* with smaller cell diameters can use 1-layer screens with the same aperture size. This may be because *D. salina* cells rapidly clogged the filter pores by producing sticky extracellular polymeric substances (EPS), which impeded further permeation. The salinity of SP is about 1.3%, which creates osmotic pressure and induces *D. salina* to protect itself from salt damage. EPS production is an additional mechanism to cope with salinity stress [32,33].

The physical properties of the screen may also affect microalgae’s escape. The actual pore sizes may differ because of screen irregularities, such as cracks or defects. These irregularities can create larger openings. Furthermore, the screen pores may change in shape due to factors such as pressure, temperature, and humidity, which enable microalgae to pass through. This agrees with the observation in Section 3.1 that the underflow proportion of *Coelastrella* sp. cultured in a single layer of 2000 mesh screen increased sharply after 12 days. The hydrodynamic mechanism of microalgal motion may also enable microalgae to escape. Most microalgae swim in three-dimensional complex trajectories that allow them to interact with their environment. The non-uniform velocity field of a microalgal slurry in tube flow can induce microalgal cell migration, which can generate pressure gradients that compel the microalgae to alter their speed and direction of movement, allowing them to cross narrower screen pores [34].

### 3.3. Effects of MSGR Diluted by Soil Extract on Microalgae

#### 3.3.1. Biomass Concentration and Photosynthetic Pigments

**Figure 3:**
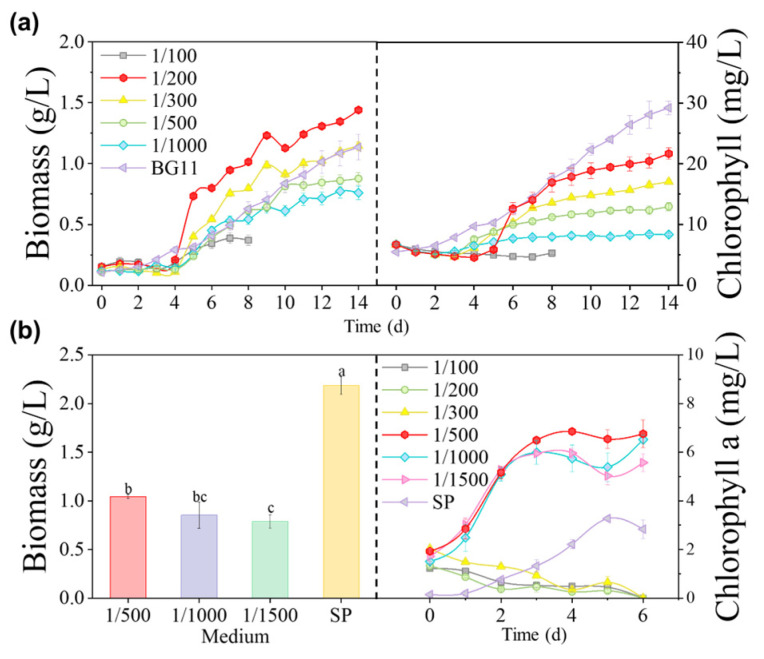
Choice of MSGR addition ratios. The effect of adding different volume ratios of MSGR to soil extracts (*V*_MSGR_/*V*_SE_) on the biomass concentrations and chlorophyll contents of (**a**) *Coelastrella* sp. and (**b**) *S*. *subsalsa*. Among them, biomass data annotated with different letters in (**b**) have a statistically significant difference by Duncan’s test at *p* < 0.05.

**Figure 4:**
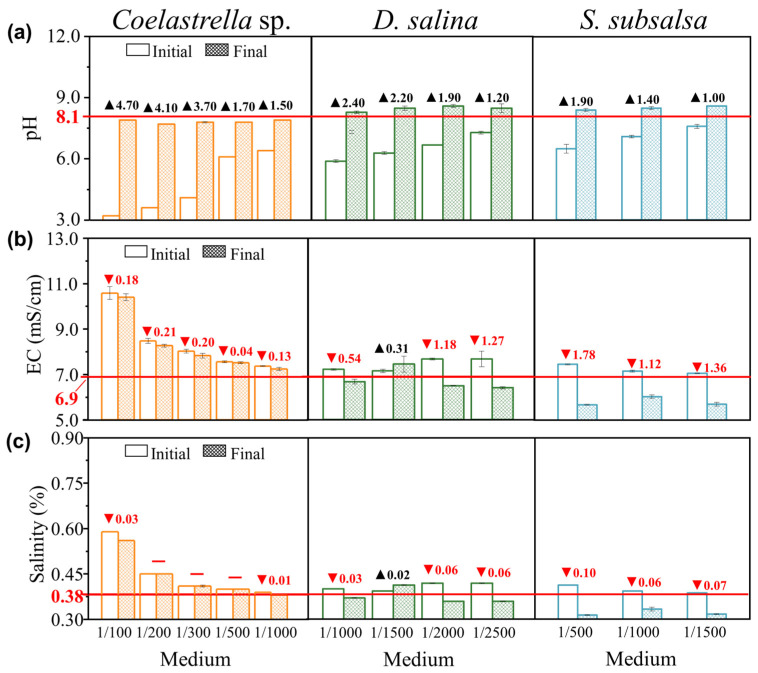
The changes in (**a**) pH, (**b**) electrical conductivity (EC), and (**c**) salinity before and after adding different volume ratios of MSGR to soil extracts (*V*_MSGR_/*V*_SE_) when culturing *Coelastrella* sp., *D*. *salina,* and *S*. *subsalsa*. The label “initial” means before cultivation, and “final” means after cultivation. The red arrows and numerical values represent decreases, while black arrows and numerical values represent increases. The values corresponding to the red horizontal lines represent the pH, EC, and salinity of the pure soil extract.

Figure 3a shows that *Coelastrella* sp. grew in diluted 1/200 MSGR, reaching the highest biomass concentration of 1.44 g/L, which was a 30% increase over the concentration of 1.13 g/L attained in BG11. However, microalgae in diluted 1/100 MSGR with higher nutritional content had already died on the eighth day. The high-concentration wastewater may have darkened the medium and blocked light transmission, adversely affecting the growth of algal cells. Nutrient limitation constrained growth in diluted 1/500 and 1/1000 MSGR. The biomass concentration of *Coelastrella* sp. remained stable for the first four days because MSGR acidified the culture medium and microalgae needed time to adjust to pH changes. Thereafter, on the fifth day, *Coelastrella* sp. entered its rapid growth phase—especially in diluted 1/200 MSGR, where it increased six-fold within five days and reached a final pH of 7.7 (Figure 4a)—demonstrating its pH adaptability. Thus, diluted 1/200 MSGR could be considered to be an effective nutrient source for *Coelastrella* sp. to improve saline–alkali soil.

Figure S5a shows the temporal changes in the biomass concentration of *D. salina*. In diluted MSGR, *D. salina* underwent a long adaptation phase and entered a logarithmic growth phase after 14 days, with a marked increase in biomass. This may reflect its adjustment to the acidic environment induced by MSGR. At a dilution of 1/2000 MSGR, the highest biomass concentration was 1.48 g/L, close to the 1.49 g/L achieved in SP. The optimal N/P ratio for microalgae to grow is around seven, whereas MSGR has a low phosphorus availability, which might limit microalgae’s growth under certain conditions. Adding phosphate to anaerobic digestate can increase microalgal growth nearly two-fold [35]. Microalgae can grow better in organic media under mixotrophic or heterotrophic conditions than in inorganic media under photoautotrophic conditions [36]. However, *D. salina* was isolated from soil and had not been acclimatized to using organic carbon from MSGR as a sole or additional carbon source. This might explain why its biomass concentration and productivity were lower than those of SP. In contrast, another species, *Coelastrella* sp., displayed significantly better performance in organic media under similar conditions. Indeed, despite being sourced from the same soil, *Coelastrella* sp. outperformed *D. salina* due to its better adaptive qualities. It likely has a superior ability to utilize organic carbon or demonstrates greater proficiency in thriving under MSGR-enriched conditions compared to *D. salina*. Its improved physiological attributes, such as efficient metabolic pathways for organic matter degradation and potentially superior enzyme systems, contribute to increased growth rates and biomass accumulation in organic cultures. Genetic differences between these two species may dictate their distinct responses to environmental transitions, like the shift from inorganic to organic carbon sources. Furthermore, even though they coexist in the same habitat, they likely occupy unique ecological niches, with evolved strategies that, when replicated in laboratory conditions, demonstrate varying degrees of adaptation to organic media. Undoubtedly, further research is necessary to confirm these observations. Moreover, when the MSGR dilution decreased from 1/1000 to 1/2000, the average specific growth rate of *D. salina* remained around 0.13 d^−1^, with little change. This implied that the nutrient concentration in MSGR was adequate for the growth at 1/2000 dilution. The specific growth rate also implied that MSGR did not inhibit the growth of *D. salina*. But this biomass is much lower than that of *D. salina* cultured with SP mentioned in Section 3.1. It is speculated that the growth status of the initially inoculated algae was not robust, which affected their final biomass and also caused their cultivation time of up to 20 days.

The final harvested biomass concentrations of *S. subsalsa* are shown in Figure 3b. The optimal biomass concentration was 1.04 g/L in 1/500 MSGR. However, this was still much lower than the 2.19 g/L achieved in the SP medium. In this study, we used raw MSGR to reduce the cultivation cost. However, organic matter in the media stimulated bacterial and zooplankton proliferation, which competed with microalgae for nutrients and caused algal cell decomposition and death. The growth of *S. subsalsa* in SE with MSGR was significantly lower than in SP, which is an inorganic medium rich in macro- and micronutrients, with a total N/P mass ratio of five. This ratio matches the elemental composition of microalgal cells and is optimal for their growth. On the other hand, MSGR contains complex organics, complexes, and precipitates that are not readily available for microalgae, and often have the N/P ratio deviating from five.

Photosynthetic pigment content and composition in algal cells indicate photosynthetic activity and cell growth to a certain degree [37]. Chlorophyll content variation was consistent with biomass concentration variation when MSGR served as the nutrient source for *Coelastrella* sp. (Figure 3a). An adaptation period occurred at the beginning of cultivation, which was prolonged with an increasing MSGR addition ratio. The rise in chlorophyll content mirrored photosynthesis enhancement in microalgal cells. The ratio of chlorophyll *a* to chlorophyll *b* content (Chl a/Chl b) varied marginally throughout the growth of *Coelastrella* sp., remaining at about 5.35 (Figure S6a). This ratio typically represents the light-harvesting capacity related to the PS II photochemical rate. Adding MSGR for cultivating *Coelastrella* sp. did not alter chlorophyll composition significantly. The ratio of carotenoids to chlorophyll content (Caro/Chl) of *Coelastrella* sp. in MSGR changed as shown in Figure S6b. Carotenoids are protective functional pigments against antioxidant stress in microalgal cells that assist chlorophyll in capturing and transferring energy, and shield chloroplasts from excessive light absorption by filtering excess light. An increase in Caro/Chl implies a decrease in the activity of complementary photopigment protein complexes and the PS II system [38]. Caro/Chl rose markedly in the initial stage of cultivation with MSGR addition, coinciding with the time when *Coelastrella* sp. acclimatized to the pH. Caro/Chl then dropped and gradually stabilized, indicating that *Coelastrella* sp. exhibited high environmental adaptability to MSGR addition, which is crucial for ameliorating saline–alkali soil.

Figure S5a shows that MSGR enhanced chlorophyll accumulation in *D. salina*. The maximum chlorophyll content was 37.32 mg/L with 1/2000 diluted MSGR, lower than the 53.92 mg/L with SP. The trend mirrored that of biomass concentration. Chl a/Chl b varied markedly in the initial period of *D. salina* growth following exposure to 1/2000 and 1/2500 diluted MSGR, reflecting a change in chlorophyll composition that affected the photoreaction phase and corresponding to microalgal acclimatization (Figure S7a). After day eight, the ratio leveled off, indicating *D. salina* adaptation to the MSGR-enriched environment and a consistent Chl a/Chl b synthesis among daughter cells. Figure S7b depicts Caro/Chl changes in *D. salina* with MSGR dilutions. The MSGR groups showed a continuous increase in Caro/Chl, implying that the self-protection system of cells was impaired. As MSGR dilution decreased, Caro/Chl slightly rose, indicating that *D. salina* increased its carotenoid content to cope with nutrient stress, which is crucial for soil improvement.

Chlorophyll is the main component of the photosynthetic system of cyanobacteria. Figure 3b shows how chlorophyll *a* levels in *S. subsalsa* changed over time in different dilutions of MSGR. *S. subsalsa* grew fast in the first three days, with the longest logarithmic growth phase and the highest chlorophyll *a* concentration (6.75 mg/L) in 1/500 diluted MSGR. Chlorophyll concentration reflects the biomass production potential of photosynthetic autotrophic microalgae, which also matched the highest biomass concentration in 1/500 MSGR. Considering specific algal species adaptability, it is noteworthy that while *S. subsalsa* performed optimally under certain MSGR conditions, other native species demonstrated distinct advantages in different environments. Growth curves revealed that *Coelastrella* sp. and *D. salina* were particularly well-adapted to low percentages of wastewater, showing enhanced biomass production. Conversely, when cultivated under wastewater conditions, *S. subsalsa* displayed reduced chlorophyll *a* content, hinting that algal species sourced from local saline soils might be more suitable for soil extract and wastewater culture compared to commercially obtained Spirulina. This aligns with previous research [39], which established that locally occurring species often possess higher activity and environmental adaptability than those purchased.

However, Figure 3b reveals an interaction between biomass and chlorophyll *a* concentration as cells were exposed to various MSGR concentrations and SP medium environments. While the addition of MSGR at dilutions of 1/500, 1/1000, and 1/1500 led to significantly elevated chlorophyll *a* levels compared to cultivation in SP medium alone, the corresponding biomass concentrations were paradoxically lower. This inconsistency in biomass accumulation can be elucidated by several interrelated factors. An upsurge in chlorophyll *a* synthesis could indicate a cellular strategy that prioritizes enhancing photosynthetic efficiency, inadvertently dampening cell proliferation and thus influencing biomass growth negatively [40]. Furthermore, while MSGR supplies essential nutrients, it may disrupt the nutrient balance within the medium, thereby impeding biomass accumulation due to imbalances. Lastly, cells need time to acclimate to new environmental conditions, during which they adjust their metabolic pathways for optimal survival and growth, potentially leading to temporary decreases in biomass production [41]. To fully comprehend these phenomena, comprehensive investigation is needed, including studying additional biochemical indicators, scrutinizing cellular metabolic pathways, and evaluating cell viability and survival rates across varying MSGR concentrations.

In brief, distinct microalgae species exhibit unique responses in growth and photosynthesis when grown in organic-rich MSGR, with *Coelastrella* sp. showing superior adaptability and carbon utilization. *D. salina* and *S. subsalsa* experience limitations due to medium complexity and competition. Therefore, future work should focus on understanding metabolic regulation under varying conditions and improving biomass yield and photosynthesis through controlled culture settings.

#### 3.3.2. Microalgae Cultivation Effects on Physical and Chemical Indicators

Figure 4a shows the pH before and after culturing *Coelastrella* sp., *D. salina,* and *S. subsalsa*. The medium pH affects the growth, metabolism, and ion uptake of microalgae, and influences physiological parameters such as cell membrane permeability, cell morphology, and growth cycles [42]. Due to the strong acidification of MSGR, the pH of the culture medium will be reduced to between 3.2 and 6.4 after addition. Eukaryotic microalgae convert extracellular HCO_3_^−^ into CO_2_ within intracellular vesicles via a catalytic mechanism involving carbonic anhydrase, which is then fixed by Rubisco, a process that concurrently consumes H^+^, thereby increasing intracellular OH^−^ concentrations. To maintain intracellular pH homeostasis, the microalgae must uptake H^+^ from the external environment for neutralization reactions. In the growth medium, as H^+^ is continually consumed, its concentration naturally declines, thus causing the pH value to rise [43]. As a result, the medium pH increased by 1.5–4.7 after culturing *Coelastrella* sp. in the experimental group with MSGR, stabilizing at around 7.8 but still lower than the pH of pure soil extract, which met the pH < 8.5 requirement for non-alkaline soils. Moreover, the culture medium becomes alkaline when microalgae assimilate nitrate. The main nitrogen source in the BG11 culture medium was nitrate, which accounted for the high pH of BG11 of up to 10.6, which was clearly unsuitable for improving saline–alkali soil. These results show that *Coelastrella* sp. can survive in a pH range of 3.2 to 10.6 and achieve high biomass concentrations, exhibiting excellent acid–base adaptability, which is of great significance for its application in saline–alkali soil improvement. The pH change with *D. salina* was similar to that with *Coelastrella* sp. The medium pH increased by 1.2–2.4 after culturing microalgae in the experimental group with MSGR. The cultivation of *D. salina* and *S. subsalsa* in 1/2000 diluted MSGR also showed a stable pH of 8.6.

The EC and salinity change in the medium reflects the salt ion concentration change in the solution (Figure 4b,c). For *Coelastrella* sp., the EC increased significantly after the addition of MSGR. Due to the limitation of shorter cultivation time, the EC changed relatively little before and after cultivation. When MSGR was diluted 1/200, the EC changed with a maximum decrease of 0.21 mS/cm, and the pH stabilized at 7.7, achieving a dual improvement in salinity and alkalinity. The result suggests that this dilution can be used for improving saline–alkali soil with *Coelastrella* sp. The cultivation of *D. salina* in 1/2000 diluted MSGR also showed an EC decrease of 1.18 mS/cm, and a salinity decrease of 0.06% (Figure 4b), indicating that 1/2000 MSGR can serve as a nutrient source for *D. salina*. Moreover, both EC and salinity increased in 1/1500 diluted MSGR. This may be due to the partial cell death during the adaptation process of this group, resulting in EPS release and Na^+^ adsorption and enrichment, leading to the salt return phenomenon. This strategy protects the cells and elevates the ion concentration in the medium. This is consistent with the observation in Section 3.2 that cells secrete EPS to clog the screen. The medium of 1/500 diluted MSGR resulted in a final pH of 8.4. The EC decreased by 1.78 mS/cm, and the salinity decreased by 0.10%. Moreover, the high protein content of *S. subsalsa* can increase the soil organic matter in a short time when applied to saline–alkali soil, which has great potential for improving soil fertility.

**Figure 5:**
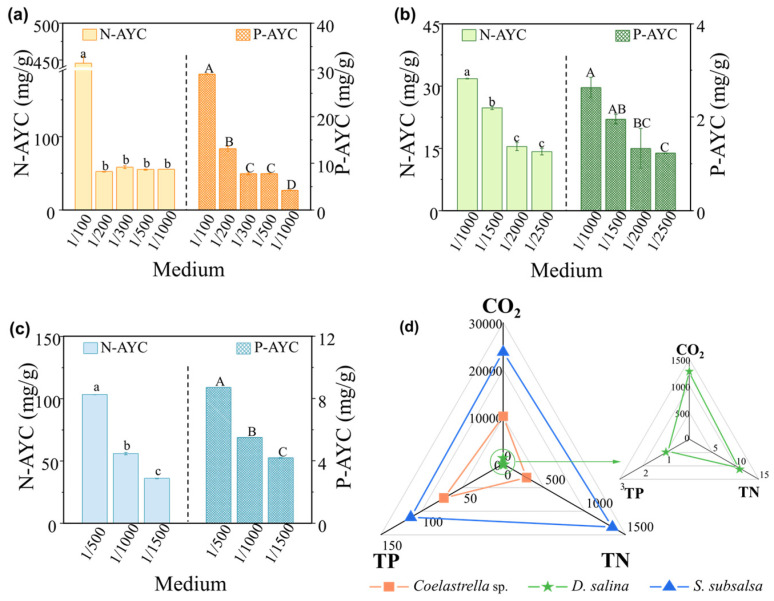
The average yield coefficients of total nitrogen (N-AYC) and total phosphorus (P-AYC) for (**a**) *Coelastrella* sp., (**b**) *D*. *salina*, and (**c**) *S*. *subsalsa* in SE supplemented with MSGR in different volume ratios (*V*_MSGR_/*V*_SE_). (**d**) Predicted rates of carbon dioxide, total nitrogen, and total phosphorus (kg/ha/year) assimilation by *Coelastrella* sp., *D*. *salina,* and *S*. *subsalsa* after underflowing and entering the soil pond of eco-farms, using the selected optimal screen and MSGR addition ratios. Data followed by different letters (a, b, c or A, B, C, and D) have a statistically significant difference by Duncan’s test at *p* < 0.05.

Microalgae can use nitrogen and phosphorus from wastewater to grow and synthesize essential substances. Figure 5a–c shows how three species of microalgae assimilated these nutrients in soil extract culture media with wastewater added. Morales-Amaral et al. [44] found that when the culture medium had enough nutrients and a balanced nitrogen–phosphorus ratio, microalgae had an N-AYC/P-AYC ratio close to 12. *Coelastrella* sp. had a low N-AYC value of 52.22 mg/g in 1/200 MSGR because it produced more biomass than the others. *D. salina* had similar N-AYC/P-AYC ratios of around 12 in all media with different MSGR dilutions, matching its consistent biomass concentration and specific growth rate. *S. subsalsa* achieved an optimal N-AYC/P-AYC ratio of approximately 12 in 1/500 MSGR and also grew best in this medium. 

Higher addition ratios of MSGR resulted in higher concentrations of ammonia nitrogen. Comparing the assimilation rate of TN and NH_3_-N in Figure S8a,c, NH_3_-N was the predominant form of nitrogen assimilated by microalgae. Ammoniacal nitrogen is generally an efficient source of nitrogen for microalgal growth. However, high concentrations of ammonia nitrogen have been shown to affect microalgal growth in various ways. They can inhibit nitrate reductase activity and alter cellular nitrogen metabolism [45], as well as reduce microalgal biomass and survival [46]. The tolerance of different microalgal species to ammonia nitrogen ranges from 25 to 1000 μmol/L. In our previous research, we found that ammonia nitrogen at 70.71 mg/L in anaerobically digested effluent from kitchen waste inhibited the growth of *Golenkinia* sp. and *Chlorella sorokiniana* [24]. This might explain why *D. salina* and *S. subsalsa* did not grow well in wastewater with a high addition ratio of nutrient concentration, despite having sufficient nitrogen and phosphorus. In contrast, *Coelastrella* sp. showed a relatively high tolerance to a high ammonia nitrogen concentration of 184.10 mg/L in 1/100 MSGR. This suggests that *Coelastrella* sp. is a suitable candidate for saline restoration using microalgae.

By examining the effects of pH, EC, and salinity on three microalgae species, the relationship between microalgae growth and environmental conditions was studied in depth. Moreover, the research demonstrated how these microalgae effectively utilize nitrogen and phosphorus nutrients from wastewater. The findings highlighted the resilience of *Coelastrella* sp. to high ammonia nitrogen concentrations, making it a promising candidate for saline soil restoration efforts. However, it also pointed out the potential negative impacts of elevated ammonia nitrogen levels on certain species such as *D. salina* and *S. subsalsa*. These discoveries enrich our understanding of microalgal ecological adaptation mechanisms.

### 3.4. Nutrient Assimilation and Economic Benefits in Microalgal Eco-Farms

According to the previously mentioned microalgal eco-farm, it was assumed that each soil pond in a single remediation module of the eco-farm would be 1 m in length and width and 0.2 m in depth. Based on the underflow proportion corresponding to the optimal screens and nutrient assimilation capacity corresponding to the optimal dilution ratio of MSGR for three microalgal species, the theoretical assimilation values of nutrients were derived. The nutrient assimilation efficiency of the whole microalgal eco-farm (*X*AE_W_) was calculated according to the following formula:

(12) $$XAE_{W}=\frac{\left(\delta \mathrm{DM}/\delta t\right)X\text{-}\mathrm{AYC}}{\mathrm{FP}}T\eta_{T}A\eta_{A}$$

where *X* stands for a particular nutrient, being carbon dioxide (CO_2_), TN, or TP. δ*DM*/δ*t* is the biomass productivity of each cultivation period. Based on the typical molecular formula (CO_0.48_H_1.83_N_0.11_P_0.01_), the theoretical C-AYC is 1.83 g CO_2_/g dry mass of biomass. FP is the footprint of a single eco-farm module. *T* and *A* are the time and area of the whole restoration process, and *η_T_* and *η_A_* are the correction factors for time and area. Considering the adverse impact of winter temperature on the harvesting process and the safety margin (1 m horizontally and vertically) between various remediation modules, *η_T_* and *η_A_* were set to 75% and 50%, respectively.

When considering the *T* and *A* efficiencies, the predicted values of carbon dioxide assimilation by these three species from the soil pond of eco-farms by underflowing the screen reached 10,093, 1257, and 23,737 kg/ha/year, significantly contributing to greenhouse gas reduction and promoting carbon neutrality. Moreover, their potential nitrogen assimilation capacities reached 288, 11, and 1340 kg/ha/year, while phosphorus assimilation reached 72, 1, and 113 kg/ha/year, respectively (Figure 5d). This can notably reduce agricultural dependence on chemical fertilizers, enhancing soil health and mitigating non-point source pollution. 

This underflow leakage of microalgae into the soil is not only beneficial in increasing soil organic matter but also highlights the importance of integrating different types of microalgae, such as nitrogen-fixing cyanobacteria and non-nitrogen-fixing species. Nitrogen-fixing naturally enhances soil fertility by converting atmospheric nitrogen, thereby reducing external input needs. On the other hand, non-nitrogen-fixing microalgae effectively utilize available nutrients in wastewater, playing a critical role in nutrient removal and biomass production.

Furthermore, having detailed the nutrient assimilation capabilities of microalgae in ecological farms, it is essential to consider the economic and environmental benefits that this groundbreaking approach brings to the table. It is pivotal to recognize the economic and environmental advantages of utilizing wastewater like MSGR as a nutrient source in microalgal cultivation. By leveraging this resource, the need for purchasing and supplementing essential nutrients, chiefly nitrogen and phosphorus, is substantially reduced. This approach not only diminishes costs but also opens up opportunities for generating revenue through the production of high-value products such as biofuels, feed additives, or other byproducts. The utilization of wastewater enables microalgae to effectively uptake and transform these nutrients, reducing the discharge of wastewater, consequently improving water quality and mitigating eutrophication issues. When conducting field saline–alkali soil tests in the future, brackish water near the land will be used to replace fresh water, thus greatly reducing the cost of fresh water.

The price of 2000 mesh or 5000 mesh screens is about USD 80,000 or USD 160,000, respectfully, per hectare, so for *Coelastrella* sp., *D. salina,* or *S. subsalsa*, the total price of screens would be about USD 80,000–480,000/ha. Considering the different geographical locations, one ha of the soil will cost about USD 80,000–150,000 to purchase. The screen and medium will be recycled, and only fresh MSGR will need to be replenished after each harvest, so these investments in the infrastructure phase are almost one-offs. Despite the substantial initial infrastructure investments, the long-term profitability of the project is promising given the multifaceted benefits and potential yields derived from wastewater utilization as discussed earlier. Calculated at USD 15,000/ton biomass, the microalgal biomass of 40–70 ton/ha/year will result in benefits of USD 600,000–1,050,000/ha/year. If further considering the high-value biological products processed from biomass, such as biodiesel priced at USD 1.3–1.5/kg, the benefits will completely outweigh the investment in the construction in just a few years.

Both *Coelastrella* sp. and *D. salina* exhibited similar final biomass concentrations (1.44 and 1.48 g/L) (Table S7). However, the growth cycle of *Coelastrella* sp. was shorter and it showed better biomass productivity above the screen. In addition, due to the underflow proportion of *D. salina* being much lower than that of *Coelastrella* sp., the biomass productivity entering the soil was about only 1/10 as much as for *Coelastrella* sp. Therefore, by comprehensively comparing the two types of algae independently screened from the soil, *Coelastrella* sp. was superior to *D. salina* in terms of biomass harvested and soil organic matter added. For the purchased *S. subsalsa*, because it had the shortest growth cycle of only six days and the highest underflow proportion, its biomass productivities above the screen and in the soil were higher than those of the other two algae. *Coelastrella* sp. can be considered an option for harvesting more biomass concentration, whereas the alternative of *S. subsalsa* has the advantage of rapid biomass acquisition. According to the changes in physical indicators before and after cultivation given in Section 3.3.2, compared to *Coelastrella* sp., the harvest of *S. subsalsa* carried away more ions, resulting in a greater decrease in EC and salinity. Yet, its final pH was higher than for *Coelastrella* sp. and indeed reached the “warning line” of 8.5, so it is necessary to closely monitor the changes in soil acidity and alkalinity during the application of *S. subsalsa* to achieve better soil improvement. Based on these comparative analyses, it becomes evident that each microalgal species offers unique advantages, thereby suggesting tailored application strategies for different ecological farm configurations or specific soil remediation needs.

## 4. Conclusions

This study explored the potential of specific salt-tolerant microalgae species, including *Coelastrella* sp. SDEC-28, *Dunaliella salina* SDEC-36, and *Spirulina subsalsa* FACHB-351, for phycoremediation in enhancing saline–alkali soil conditions within eco-farms. Nylon screens were effectively employed to harvest microalgal biomass from the growth medium, with the underflow proportion being influenced by the motion and metabolic characteristics of microalgae. Further experiments evaluated the use of MSGR diluted at varying ratios with soil extracts as a nutrient supplement for cultivating these selected strains. The optimal MSGR addition ratio was determined based on the nitrogen and phosphorus utilization efficiency of microalgae, achieved biomass concentrations, and observed changes in the physiochemical properties of the culture medium, which simulated improvements to actual soil conditions. Through assessing algal growth dynamics and nutrient assimilation capabilities, the study substantiated the feasibility of increasing soil organic matter content through the cultivation of these microalgae. Ultimately, this work supports the rationale for establishing microalgae-based eco-farms that contribute to sustainable soil improvement.

## Data Availability

Data are contained within the article.

## Supplementary Materials

The following supporting information can be downloaded at: https://www.mdpi.com/article/10.3390/microorganisms12040676/s1, Figure S1: Decision model for screen selection; Figure S2: Choice of screen aperture and number of layers. The biomass concentrations of (a) *S. subsalsa* cultured in different numbers of screen layers, with 2000 mesh or 5000 mesh screens. (b) The scores of each alternative for *Coelastrella* sp., *D*. *salina,* and *S. subsalsa* were obtained from AHP analysis. *S*. *subsalsa* was cultured using a standard medium (SP medium). “Control” indicates the condition without any screen. The “1-layer”, “2-layer”, and “3-layer” labels indicate the use of 1, 2, and 3 layers of screens, respectively. The label “in” indicates the part inside the screens, and “ex” indicates the part that underflows the screens; Figure S3: Micrographs of 2000 mesh and 5000 mesh screens for the control and *Coelastrella* sp. SDEC-28, *D*. *salina* SDEC-36 and *S*. *subsalsa* FACHB-351 before and after cultivation; Figure S4: Micrographs of *Coelastrella* sp., *D*. *salina* and *S*. *subsalsa*. (a) *Coelastrella* sp. in BG11 (left) and BG11 with a salinity of 4% (right); (b) *D*. *salina* fixed with alcohol (left) and in natural state (right); and (c) the helicoid shape (left) and the breaks formed by fractures (right) of *S*. *subsalsa*. (d) Diagram illustrating the relationship between microalgae characteristics and screen properties, with red text indicating factors promoting microalgae escape and blue text representing preventive measures; Figure S5: Choice of MSGR addition ratios. The effect of adding different volume ratios of MSGR to soil extracts (*V*_MSGR_/*V*_SE_) on the biomass concentrations and chlorophyll contents of (a) *D*. *salina*. (b) The biomass productivities (*P*) and average specific growth rates (*μ*) of these three microalgae in different media. Biomass productivity data annotated with different letters have a statistically significant difference by Duncan’s test at *p* < 0.05; Figure S6: The variation of (a) chlorophyll *a* to chlorophyll *b* ratio (Chl a/Chl b) and (b) ratio of carotenoids to chlorophyll (Caro/Chl) for *Coelastrella* sp. in BG11, and in SE supplemented with MSGR at different volume ratios (*V*_MSGR_/*V*_SE_ ranging from 1/1000 to 1/100); Figure S7: The variation of (a) chlorophyll *a* to chlorophyll *b* ratio (Chl a/Chl b) and (b) ratio of carotenoids to chlorophyll (Caro/Chl) for *D*. *salina* in SP, and in SE supplemented with MSGR at different volume ratios (*V*_MSGR_/*V*_SE_ ranging from 1/1000 to 1/100); Figure S8: The assimilation rate (*AR*) of *Coelastrella* sp., *D*. *salina* and *S*. *subsalsa* for (a) TN, (b) TP, and (c) NH_3_-N in SE supplemented with MSGR at different volume ratios (*V*_MSGR_/*V*_SE_); Table S1: Criteria and alternatives; Table S2: Pairwise comparison matrix; Table S3: Pairwise comparison matrix for *C*_1_ applied to *Coelastrella* sp.; Table S4: Pairwise comparison matrix for *C*_2_ applied to *Coelastrella* sp.; Table S5: Pairwise comparison matrix for *C*_3_ applied to *Coelastrella* sp.; Table S6: Priority vectors for the AHP applied to *Coelastrella* sp.; Table S7: The biomass of *Coelastrella* sp., *D. salina* and *S. subsalsa* with the optimal screen and MSGR dilution. References [47,48] are cited in the Supplementary Materials.
