# Supplementary material for: Phycoremediation Potential of Salt-Tolerant Microalgal Species: Motion, Metabolic Characteristics, and Their Application for Saline–Alkali Soil Improvement in Eco-Farms
Source: Microorganisms. 2024 Mar 28;12(4):676. doi: 10.3390/microorganisms12040676 (PMC11052205; doi:10.3390/microorganisms12040676)
Supplement: Supplementary file 1 [file microorganisms-12-00676-s001.zip › microorganisms-2912977-supplementary.pdf]

Supplementary material

Phycoremediation Potential of Salt-Tolerant Microalgal Species: Motion, Metabolic Characteristics, and Their Application for Saline–Alkali Soil Improvement in Eco-farms

Huiying Chen [1], Siteng Yu [1], Ze Yu [2], Meng Ma [1], Mingyan Liu [1] and Haiyan Pei [1,2,3,4,*]

[1] School of Environmental Science and Engineering, Shandong University, Qingdao 266237, China;
chenhuiying07@163.com (H.C.); y820099450@163.com (S.Y.);
15001956061@163.com (M.M.); 17853728873@163.com (M.L.)
[2] Department of Environmental Science and Engineering, Fudan University, Shanghai 200433, China; yz201512044@126.com
[3] Shandong Provincial Engineering Center on Environmental Science and Technology, Jinan 250061, China
[4] Institute of Eco-Chongming (IEC), Shanghai 202162, China

* Correspondence: haiyan_phyto@fudan.edu.cn

2. Materials and methods

Previous cultivations of *Coelastrella* sp. were performed in photobioreactors at 25 ± 1 °C under 45 μmol/m^2/s of 24 h light to attain healthy seed cells for further studies in BG11 medium, which is composed of (per litre of distilled water): 1.50 g $NaNO_3$, 0.04 g K_2HPO_4, 0.075 g $MgSO_4·7H_2O$, 0.036 g $CaCl_2·2H_2O$, 0.006 g citric acid, 0.006 g ferric ammonium citrate, 0.001 g EDTA-Na_2, 0.02 g Na_2CO_3, and 1 mL of A_5. A_5 trace metal solution consists of (per litre of distilled water): 2.86 g H_3BO_3, 1.86 g $MnCl_2·4H_2O$, 0.22 g $ZnSO_4·7H_2O$, 0.39 g $Na_2MoO_4·2H_2O$, 0.08 g $CuSO_4·5H_2O$ and 0.05 g $Co(NO_3)_2·6H_2O$.

D. salina and *S. subsalsa* were treated under the same conditions in the SP medium, which is composed of (per litre of distilled water): 13.61 g $NaHCO_3$, 4.03 g Na_2CO_3, 0.50 g K_2HPO_4, 2.50 g $NaNO_3$, 1.00 g K_2SO_4, 1.00 g NaCl, 0.20 g $MgSO_4·7H_2O$, 0.04 g $CaCl_2·2H_2O$, 0.04 g $FeSO_4·7H_2O$ and 1 mL of A_5.

3. Results and discussion

3.1. Effects of screen aperture size and number of layers on growth of microalgae

The aim of this study is to identify the best screen for each microalgal species (*Coelastrella* sp., *D. salina* and *S. subsalsa*). Multi-criterion decision analysis (MCDA) methodologies were adopted to resolve this problem. The criteria that can influence screen selection can be summarised as the biomass concentration inside the screen, underflow proportion, and screen price. Among the criteria, some are considered to be more important than the others. Weighting involves assigning numerical measures to each criterion according to its relative importance — these numerical measures are the "weights" [1]. Among MCDA methods, the analytic hierarchy process (AHP) was developed by Saaty [2].

Figure S1 shows the MCDA decision model for this MCDA problem. For each strain, six screens will be evaluated using three criteria. Table S1 shows a summary of the alternatives, the criteria, and the input values used in this study.

The following procedures are used in this study.

<u>(1) Assigning weights</u>

Tables S2 shows the pairwise comparison matrices. The matrices are normalized and the Consistency Ratio (CR) calculated to ensure consistency. From Table S2, the CR = 0.001 < 0.1. Therefore, the pairwise comparison is consistent and the criterion weights can be used for further decision making. The weights of the criteria are: **C_1 (Internal biomass concentration) = 0.460; C_2 (Underflow proportion) = 0.319; C_3 (Screen price)**

= **0.221**. Among the three criteria considered, the internal biomass concentration has the highest weight, followed by the underflow proportion. This implies that, based on the judgment of all authors in this article (with data from the literature, *etc.*), the best screen for microalgae has to have very high internal biomass concentration and a low underflow proportion.

(2) Assessing alternatives

Every alternative is assessed based on an individual criterion to obtain weights. One pairwise comparison matrix is constructed for every criterion; within each matrix one alternative is compared to every other alternative.

i. For *Coelastrella* sp.

According to the one-way ANOVA in SPSS, the pairwise comparison matrix for C_1 (Internal biomass concentration) for *Coelastrella* sp. is obtained as in Table S3. The matrices are normalized and the CR (0.002) calculated to ensure consistency. For C_1, the weights of ID01 to ID06 are: $\boldsymbol{W_{ID01}}$ **= 0.082**; $\boldsymbol{W_{ID02}}$ **= 0.155**; $\boldsymbol{W_{ID03}}$ **= 0.155**; $\boldsymbol{W_{ID04}}$ **= 0.155**; $\boldsymbol{W_{ID05}}$ **= 0.155**; and $\boldsymbol{W_{ID06}}$ **= 0.298**.

Similarly, Table S4 and Table S5 present the pairwise comparison matrices for C_2 (Underflow proportion) and C_3 (Screen price), for which the respective CR values are 0.024 and 0.020. For C_2, the weights of ID01 to ID06 are: $\boldsymbol{W_{ID01}}$ **= 0.043**; $\boldsymbol{W_{ID02}}$ **= 0.160**; $\boldsymbol{W_{ID03}}$ **= 0.102**; $\boldsymbol{W_{ID04}}$ **= 0.066**; $\boldsymbol{W_{ID05}}$ **= 0.250** and $\boldsymbol{W_{ID06}}$ **= 0.379**. For C_3, the weights of ID01 to ID06 are: $\boldsymbol{W_{ID01}}$ **= 0.413**; $\boldsymbol{W_{ID02}}$ **= 0.221**; $\boldsymbol{W_{ID03}}$ **= 0.083**; $\boldsymbol{W_{ID04}}$ **= 0.146**; $\boldsymbol{W_{ID05}}$ **= 0.086**; and $\boldsymbol{W_{ID06}}$ **= 0.051**.

Table S6 shows a collation of the priority vectors for the three criteria applied to *Coelastrella* sp..

For every alternative, the priority vectors are multiplied by the individual criterion weights. The sum of these products are the scores for each alternative. The alternative with the highest score is the best.

For ID01 the score is calculated as:

(0.082×0.460) + (0.043×0.319) + (0.413×0.221) = **0.143**.

The scores for the other alternatives are: $\boldsymbol{S}_{\mathbf{ID02}}$ **= 0.172;** $\boldsymbol{S}_{\mathbf{ID03}}$ **= 0.122;** $\boldsymbol{S}_{\mathbf{ID04}}$ **= 0.125;** $\boldsymbol{S}_{\mathbf{ID05}}$ **= 0.170; and** $\boldsymbol{S}_{\mathbf{ID06}}$ **= 0.269**.

The best configuration is **ID06 (5000 mesh 3-layer)**.

ii. For *D. salina*

Similarly, for *D. salina* the scores for all alternatives are: $\boldsymbol{S}_{\mathbf{ID07}}$ **= 0.152;** $\boldsymbol{S}_{\mathbf{ID08}}$ **= 0.088;** $\boldsymbol{S}_{\mathbf{ID09}}$ **= 0.181;** $\boldsymbol{S}_{\mathbf{ID10}}$ **= 0.241;** $\boldsymbol{S}_{\mathbf{ID11}}$ **= 0.187; and** $\boldsymbol{S}_{\mathbf{ID12}}$ **= 0.150**.

The best configuration is **ID10 (5000 mesh 1-layer)**.

iii. For *S. subsalsa*

Similarly, for *S. subsalsa* the scores for all alternatives are: $\boldsymbol{S}_{\mathbf{ID13}}$ **= 0.249;** $\boldsymbol{S}_{\mathbf{ID14}}$ **= 0.134;** $\boldsymbol{S}_{\mathbf{ID15}}$ **= 0.141;** $\boldsymbol{S}_{\mathbf{ID16}}$ **= 0.136;** $\boldsymbol{S}_{\mathbf{ID17}}$ **= 0.211; and** $\boldsymbol{S}_{\mathbf{ID18}}$ **= 0.128**.

The best configuration is **ID13 (2000 mesh 1-layer)**.

(3) Summary

In summary, considering the balance of three factors in the cultivation — *i.e.*, obtaining as much biomass concentration above the screen as possible, as little a

proportion of cells underflowing beyond the screen as possible, and as low a screen price as possible — 5000 mesh 3-layer, 5000 mesh 1-layer, and 2000 mesh 1-layer were optimal for soil treatment of *Coelastrella* sp., *D. salina* and *S. subsalsa*, respectively.

References

1. Triantaphyllou, E., Mann, S.H. An examination of the effectiveness of multi-dimensional decision-making methods: a decision-making paradox. *Decis. Support Syst.* **1989**, *5(3)*, 303–312.
2. Satty, T.L. How to make a decision: the analytic hierarchy process. *Eur. J. Oper. Res.* **1990**, *48(1)*, 9–26.

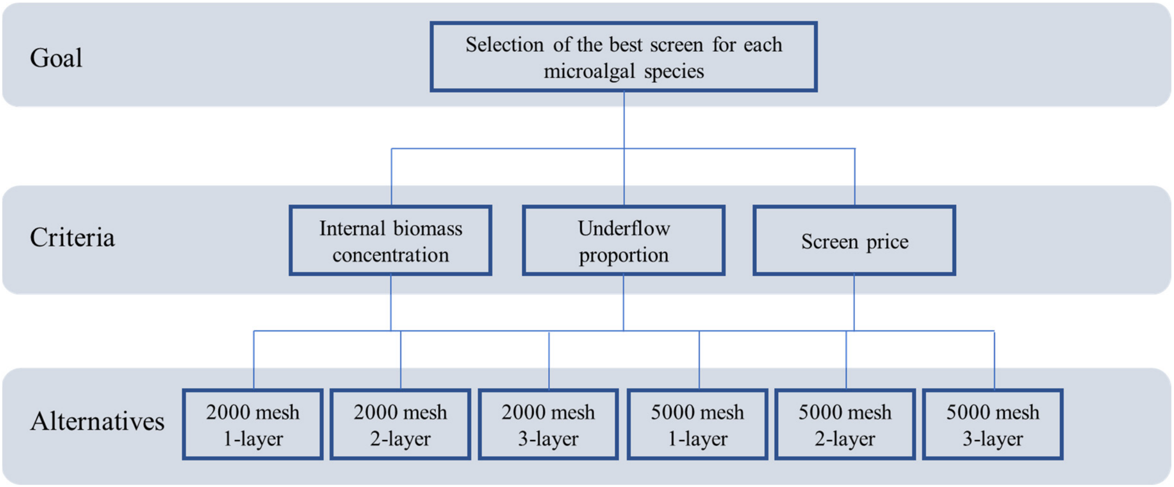

Figure S1. Decision model for screen selection.

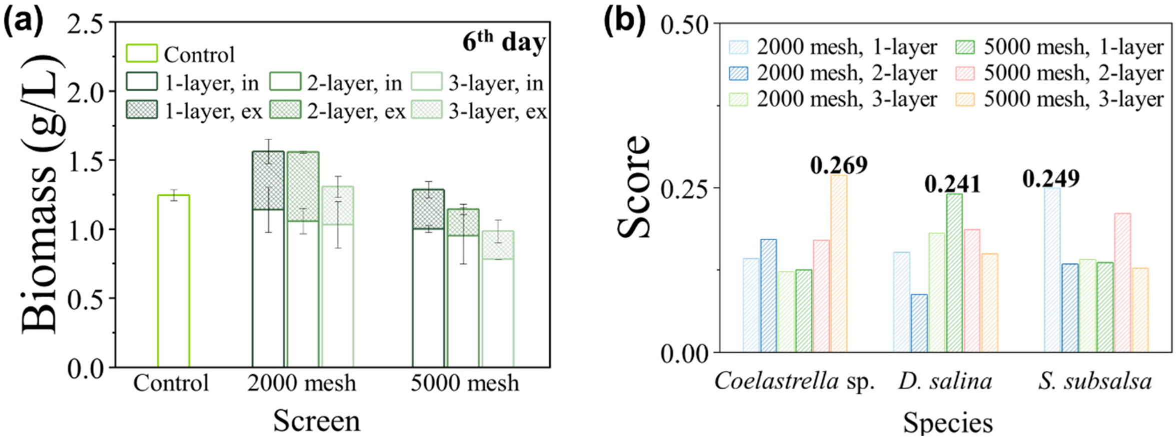

Figure S2. Choice of screen aperture and number of layers. The biomass concentrations of (a) *S. subsalsa* cultured in different numbers of screen layers, with 2000 mesh or 5000 mesh screens. (b) The scores of each alternative for *Coelastrella* sp., *D. salina* and *S. subsalsa* obtained from AHP analysis. *S. subsalsa* was cultured using standard medium (SP medium). "Control" indicates the condition without any screen. The "1-layer", "2-layer", and "3-layer" labels indicate the use of 1, 2, and 3 layers of screens, respectively. The label "in" indicates the part inside the screens, and "ex" indicates the part that underflows the screens.

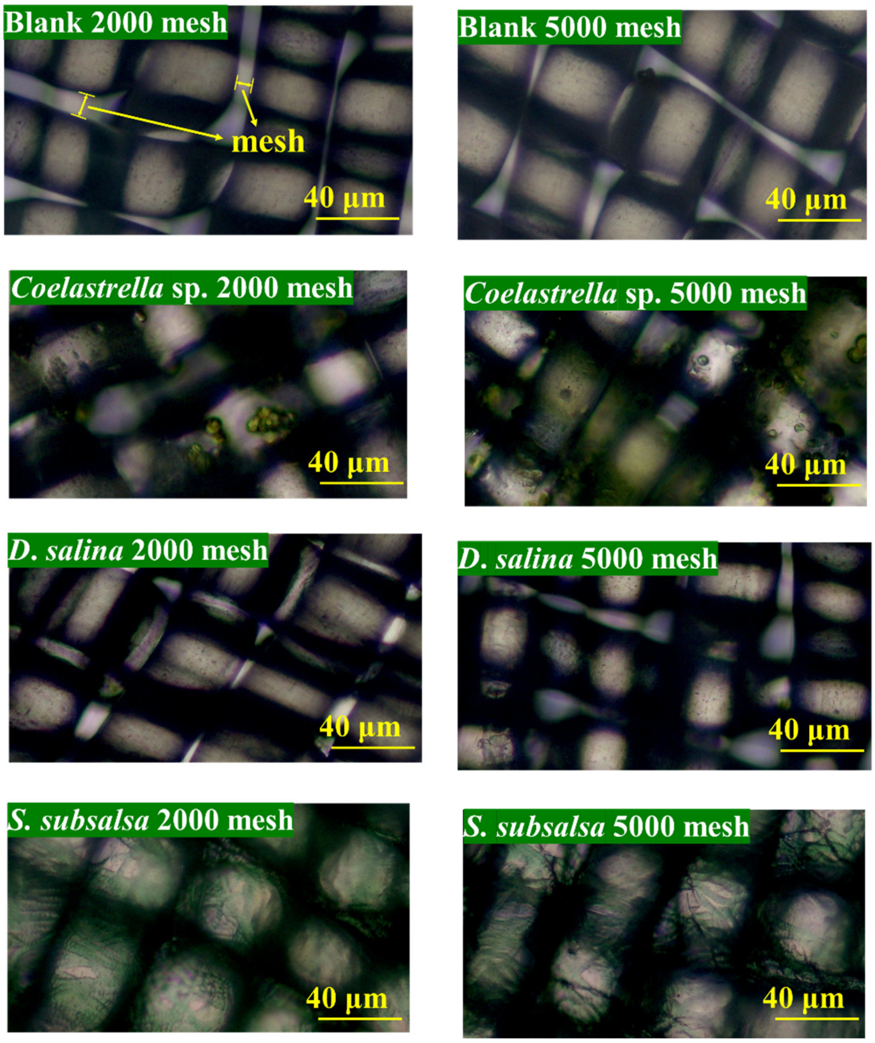

Figure S3. Micrographs of 2000 mesh and 5000 mesh screens for the control and for *Coelastrella* sp. SDEC-28, *D. salina* SDEC-36 and *S. subsalsa* FACHB-351 before and after cultivation.

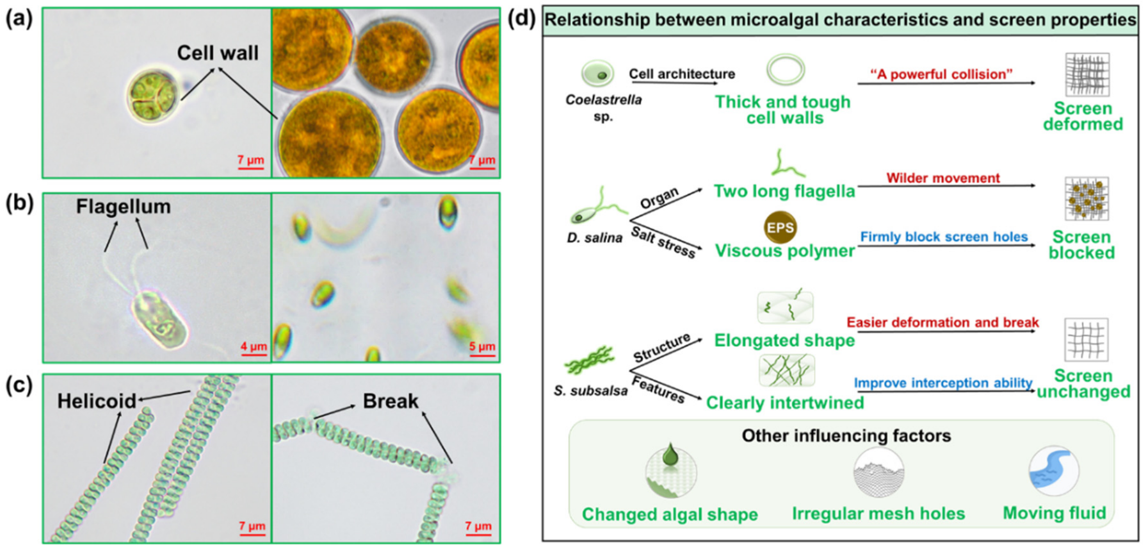

Figure S4. Micrographs of *Coelastrella* sp., *D. salina* and *S. subsalsa*. (a) *Coelastrella* sp. in BG11 (left) and BG11 with a salinity of 4% (right); (b) *D. salina* fixed with alcohol (left) and in natural state (right); and (c) the helicoid shape (left) and the breaks formed by fractures (right) of *S. subsalsa*. (d) Diagram illustrating the relationship between microalgae characteristics and screen properties, with red text indicating factors promoting microalgae escape and blue text representing preventive measures.

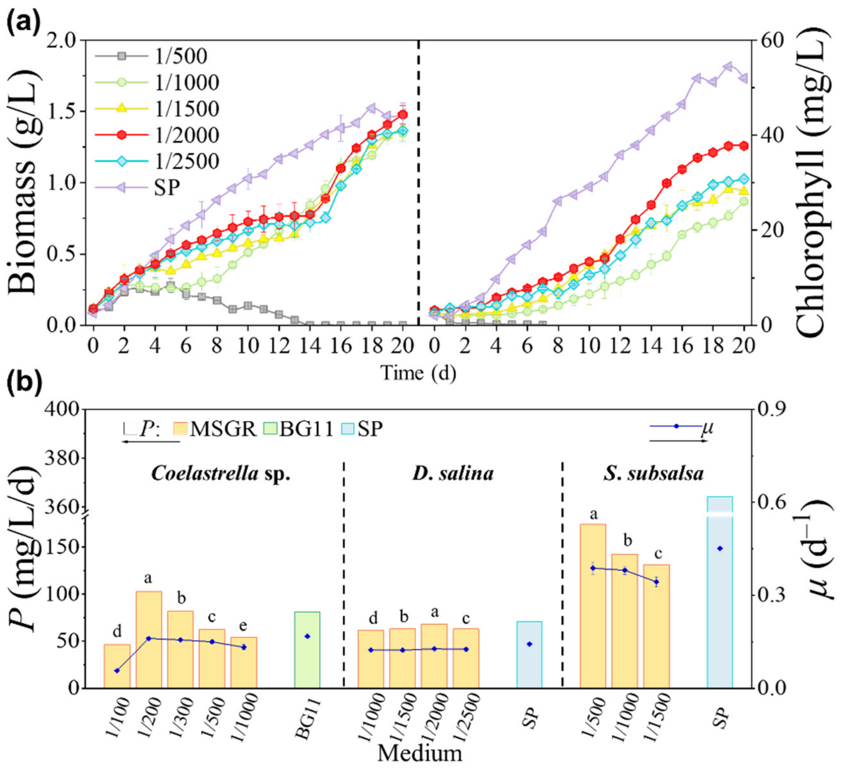

Figure S5. Choice of MSGR addition ratios. The effect of adding different volume ratio of MSGR to soil extracts ($V_{\mathrm{MSGR}}/V_{\mathrm{SE}}$) on the biomass concentrations and chlorophyll contents of (a) *D. salina*. (b) The biomass productivities (P) and average specific growth rates (μ) of these three microalgae in different media. Biomass productivity data annotated with different letters have a statistically significant difference by Duncan's test at $p<0.05$.

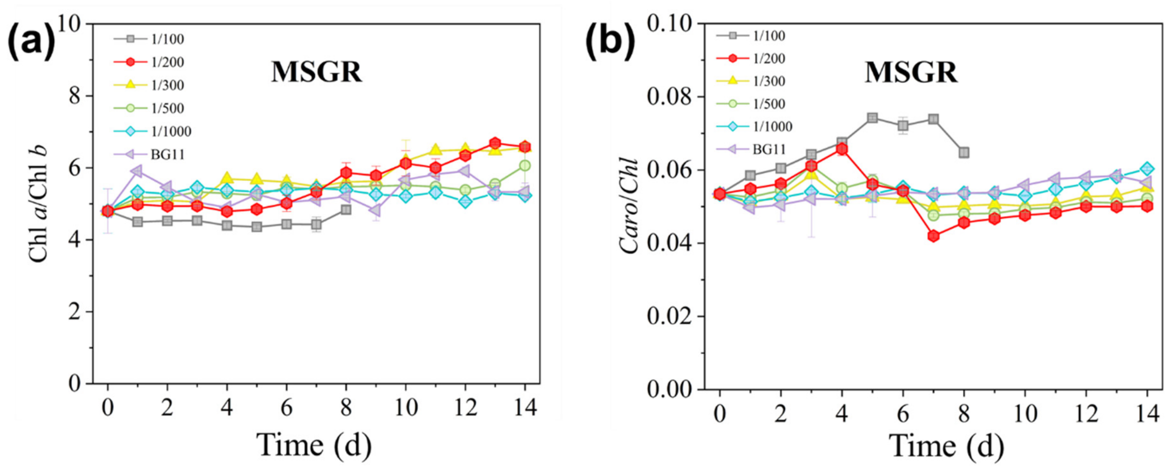

Figure S6. The variation of (a) chlorophyll *a* to chlorophyll *b* ratio (Chl a/Chl b) and (b) ratio of carotenoids to chlorophyll (Caro/Chl) for *Coelastrella* sp. in BG11, and in SE supplemented with MSGR at different volume ratios (V_{MSGR}/V_{SE} ranging from 1/1000 to 1/100).

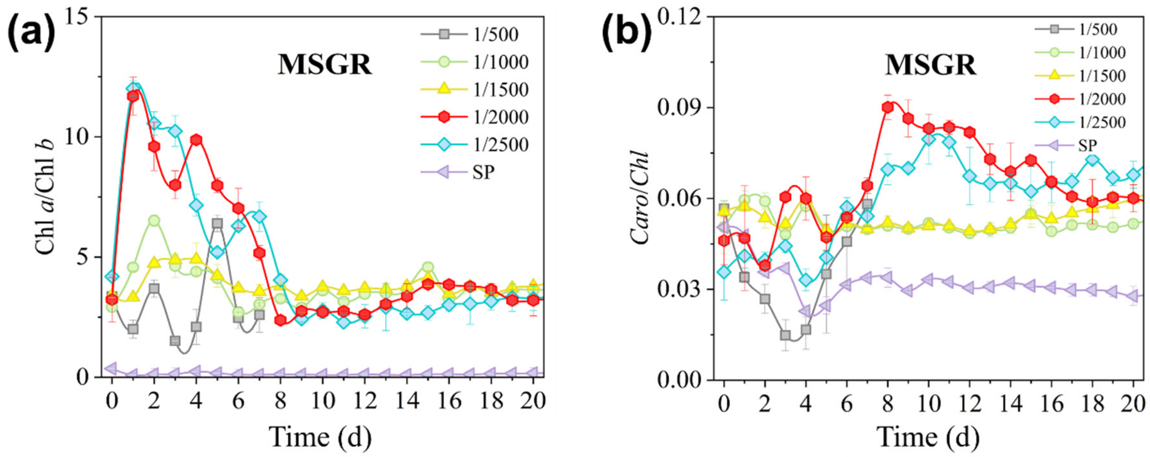

Figure S7. The variation of (a) chlorophyll *a* to chlorophyll *b* ratio (Chl a/Chl b) and (b) ratio of carotenoids to chlorophyll (Caro/Chl) for *D. salina* in SP, and in SE supplemented with MSGR at different volume ratios (V_{MSGR}/V_{SE} ranging from 1/1000 to 1/100).

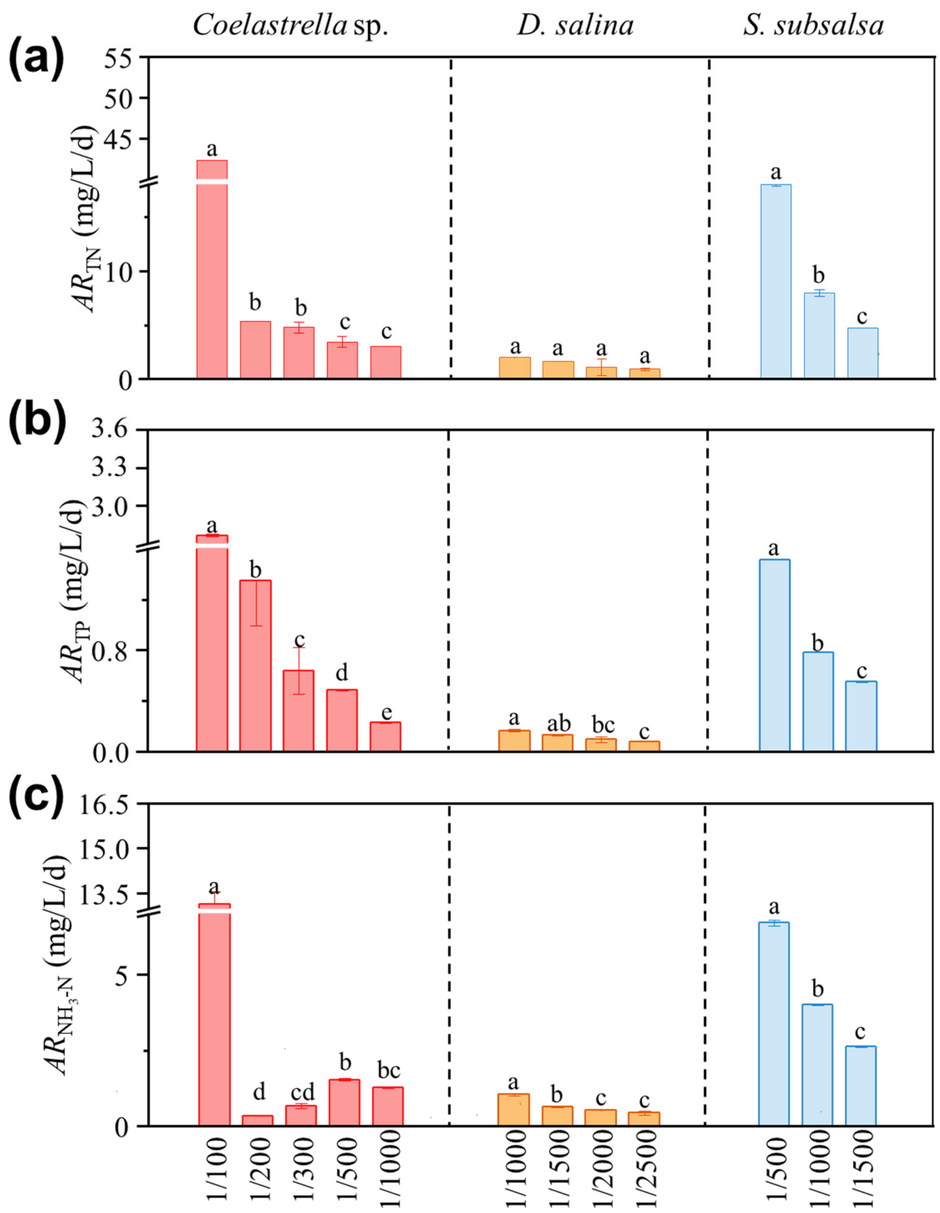

Figure S8. The assimilation rate (*AR*) of *Coelastrella* sp., *D. salina* and *S. subsalsa* for (a) TN, (b) TP, and (c) NH_3-N in SE supplemented with MSGR at different volume ratios (V_{MSGR}/V_{SE}).

Table S1. Criteria and alternatives.

Screen ID		C_1, Internal biomass concentration (g/L)	C_2, Underflow proportion (%)	C_3, Screen price ($/m^2)
Coelastrella sp.	ID01, 2000 mesh 1-layer	0.991	43.65	7.73
	ID02, 2000 mesh 2-layer	1.100	24.22	15.46
	ID03, 2000 mesh 3-layer	1.112	27.64	23.19
	ID04, 5000 mesh 1-layer	1.158	30.77	16.16
	ID05, 5000 mesh 2-layer	1.194	20.35	32.32
	ID06, 5000 mesh 3-layer	1.337	19.59	48.48
D. salina	ID07, 2000 mesh 1-layer	1.955	14.96	7.73
	ID08, 2000 mesh 2-layer	1.815	15.44	15.46
	ID09, 2000 mesh 3-layer	2.538	6.78	23.19
	ID10, 5000 mesh 1-layer	3.430	3.40	16.16
	ID11, 5000 mesh 2-layer	3.519	3.92	32.32
	ID12, 5000 mesh 3-layer	3.413	4.56	48.48
S. subsalsa	ID13, 2000 mesh 1-layer	1.141	27.07	7.73
	ID14, 2000 mesh 2-layer	1.060	32.01	15.46
	ID15, 2000 mesh 3-layer	1.032	21.10	23.19
	ID16, 5000 mesh 1-layer	1.004	22.12	16.16
	ID17, 5000 mesh 2-layer	0.954	16.72	32.32
	ID18, 5000 mesh 3-layer	0.783	20.68	48.48

Table S2. Pairwise comparison matrix.

Criterion	C_1	C_2	C_3
C_1	1	1.5	2
C_2	0.667	1	1.5
C_3	0.5	0.667	1

Table S3. Pairwise comparison matrix for C_1 applied to *Coelastrella* sp..

	ID01	ID02	ID03	ID04	ID05	ID06
ID01	1	0.5	0.5	0.5	0.5	0.333
ID02	2	1	1	1	1	0.5
ID03	2	1	1	1	1	0.5
ID04	2	1	1	1	1	0.5
ID05	2	1	1	1	1	0.5
ID06	3	2	2	2	2	1

Table S4. Pairwise comparison matrix for C_2 applied to *Coelastrella* sp..

	ID01	ID02	ID03	ID04	ID05	ID06
ID01	1	0.25	0.333	0.5	0.2	0.167
ID02	4	1	2	3	0.5	0.333
ID03	3	0.5	1	2	0.333	0.25
ID04	2	0.333	0.5	1	0.25	0.2
ID05	5	2	3	4	1	0.5
ID06	6	3	4	5	2	1

Table S5. Pairwise comparison matrix for C_3 applied to *Coelastrella* sp..

	ID01	ID02	ID03	ID04	ID05	ID06
ID01	1	0.5	0.333	0.5	0.25	0.167
ID02	2	1	0.5	1	0.333	0.25
ID03	3	2	1	2	0.5	0.333
ID04	2	1	0.5	1	0.333	0.2
ID05	4	3	2	3	1	0.333
ID06	6	4	3	5	3	1

Table S6. Priority vectors for the AHP applied to *Coelastrella* sp..

	ID01	ID02	ID03	ID04	ID05	ID06
C_1	0.082	0.155	0.155	0.155	0.155	0.298
C_2	0.043	0.160	0.102	0.066	0.250	0.379
C_3	0.413	0.221	0.083	0.146	0.086	0.051

Table S7. The biomass of *Coelastrella* sp., *D. salina* and *S. subsalsa* with the optimal screen and MSGR dilution.

Algal species	Screen	Underflow proportion (%)	MSGR dilution (V_{MSGR}/V_{SE})	Cultivation cycle (d)	Harvested biomass concentration (g/L)	Biomass productivity — in the soil (mg/L/d)	Biomass productivity — in the screen (mg/L/d)
Coelastrella sp.	5000 mesh, 3-layer	19.59	1/200	14	1.44	20.15	82.71
D. salina	5000 mesh, 1-layer	3.40	1/2000	20	1.48	2.51	71.37
S. subsalsa	2000 mesh, 1-layer	27.24	1/500	6	1.04	47.38	126.58
